# Swimming-Induced Pulmonary Edema: Evaluation of Prehospital Treatment With CPAP or Positive Expiratory Pressure Device

**DOI:** 10.1016/j.chest.2022.02.054

**Published:** 2022-03-11

**Authors:** Claudia Seiler, Linda Kristiansson, Cecilia Klingberg, Josefin Sundh, Annika Braman Eriksson, Daniel Lundeqvist, Kristofer F. Nilsson, Maria Hårdstedt

**Affiliations:** a Department of Anesthesiology and Intensive Care, Falun Hospital, Falun, Sweden; b Center for Clinical Research Dalarna-Uppsala University, Falun, Sweden; c School of Medical Sciences, Faculty of Medicine and Health, Örebro University, Örebro, Sweden; d Department of Respiratory Medicine, Faculty of Medicine and Health, Örebro University, Örebro, Sweden; e Department of Cardiothoracic and Vascular Surgery, Faculty of Medicine and Health, Örebro University, Örebro, Sweden; f Sandviken North Primary Health Care Center, Sandviken, Sweden; g Center for Research and Development, Uppsala University/Region Gävleborg, Gävle, Sweden; h Vansbro Primary Health Care Center, Vansbro, Sweden; i Department of Internal Medicine, Mora Hospital, Mora, Sweden

**Keywords:** CPAP, lung ultrasound, positive expiratory pressure device, SIPE, swimming-induced pulmonary edema, treatment, ultrasonography, LUS, lung ultrasound, MMU, mobile medical unit, NPPV, noninvasive positive pressure ventilation, PEP, positive expiratory pressure, SIPE, swimming-induced pulmonary edema, Spo_2_, peripheral oxygen saturation

## Abstract

**Background:**

Swimming-induced pulmonary edema (SIPE) occasionally occurs during swimming in cold open water. Although optimal treatment for SIPE is unknown, non-invasive positive pressure ventilation (NPPV) is an option for prehospital treatment.

**Research Question:**

Is NPPV a feasible and safe prehospital treatment for SIPE, and which outcome measures reflect recovery after treatment?

**Study Design and Methods:**

A prospective observational study was conducted at Vansbrosimningen, Sweden’s largest open water swimming event, from 2017 through 2019. Swimmers with a diagnosis of SIPE and with peripheral oxygen saturation (Spo_2_) of ≤ 95%, persistent respiratory symptoms, or both were eligible for the study. NPPV was administered on site as CPAP by facial mask or as positive expiratory pressure (PEP) by a PEP device. Discharge criteria were Spo_2_ of > 95% and clinical recovery. Four outcome measures were evaluated: Spo_2_, crackles on pulmonary auscultation, pulmonary edema on lung ultrasound (LUS), and patient-reported respiratory symptoms.

**Results:**

Of 119 treated individuals, 94 received CPAP, 24 received treatment with a PEP device, and one required tracheal intubation. In total, 108 individuals (91%) were discharged after NPPV for a median of 10 to 20 min and 11 individuals (9%) required hospital transfer. NPPV resulted in increased Spo_2_ from a median of 91% to 97% (*P* < .0001) together with improvement of six patient-reported respiratory symptoms (median numerical rating scales, 1-7 to 0-1; *P* < .0001). No significant decrease in auscultation of crackles (93% vs 87%; *P* = .508) or pulmonary edema on LUS (100% vs 97%; *P* = .500) was seen during NPPV treatment.

**Interpretation:**

NPPV administered as CPAP or via a PEP device proved feasible and safe as prehospital treatment for SIPE with a vast majority of patients discharged on site. Spo_2_ and patient-reported respiratory symptoms reflected recovery after treatment, whereas pulmonary auscultation or LUS findings did not.

## Graphical Abstract

**Figure:**
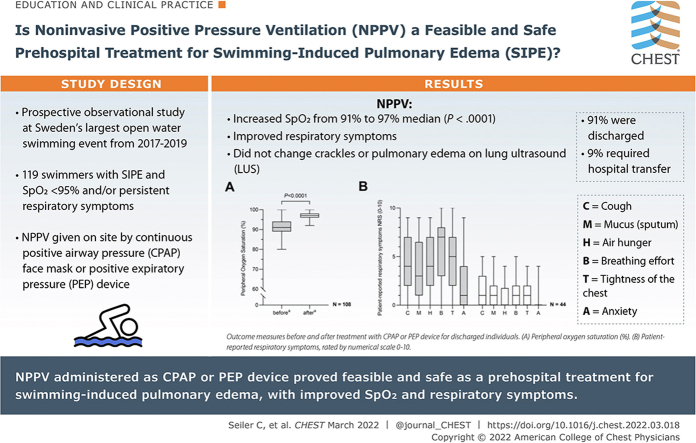

**Take-home Points**

**Study Question:** Is noninvasive positive pressure ventilation (NPPV) a feasible and safe prehospital treatment for swimming-induced pulmonary edema?

**Results:** Most patients (91%) could be discharged safely after a median of 10 to 20 min of NPPV treatment on site.

**Interpretation:** NPPV, administered as CPAP ventilation or by a positive expiratory pressure device, proved feasible and safe as prehospital treatment for swimming-induced pulmonary edema.

FOR EDITORIAL COMMENT, SEE PAGE 277

Swimming-induced pulmonary edema (SIPE) occurs in predominantly healthy individuals during swimming in cold open water.^1^ Symptoms consist of dyspnea, cough, or hemoptysis together with hypoxemia and findings of crackles on pulmonary auscultation.^1^^,^^2^ Pulmonary edema in patients with SIPE has been visualized by radiograph or CT scan, but also been confirmed in prehospital settings by point-of-care lung ultrasound (LUS).^1^, ^2^, ^3^ Although the pathophysiologic features are not understood fully, SIPE is considered a hydrostatic edema with high pulmonary capillary transmural pressure. The proposed mechanism is a combination of central pooling of blood and increased left ventricular afterload resulting from immersion in cold water, increased pulmonary capillary pressure during physical exercise, and negative intrathoracic pressures during head-out water immersion in susceptible individuals.^4^, ^5^, ^6^, ^7^, ^8^ Consequently, SIPE usually resolves spontaneously within 24 to 48 h after removal of the patient from water and rest, but occasionally may be life-threatening.^1^^,^^9^^,^^10^ The optimal strategy for treatment of SIPE is unknown. Based on pathophysiologic characteristics, noninvasive positive pressure ventilation (NPPV) could be a favorable treatment.^5^^,^^11^ Interestingly, other than a pilot study published by our research group in 2016, we could find only seven cases of patients treated with NPPV for SIPE in the literature.^9^^,^^12^, ^13^, ^14^, ^15^, ^16^, ^17^

An important aspect of prehospital care for SIPE is the growing popularity of open water swimming competitions that challenges the surrounding health care organizations.^15^^,^^18^ Vansbrosimningen is the largest open water swimming event in Sweden and attracts approximately 11,000 participants yearly. A considerable number of patients with SIPE seeking medical care during the swimming event each year, together with a 78-km distance to the nearest hospital, puts pressure on the on-site prehospital medical service.^19^ With the purpose of providing efficient treatment on site and possibly saving ambulance and hospital resources, prehospital CPAP for treatment of SIPE was implemented during Vansbrosimningen in 2013.^15^ In 2019, treatment with a positive expiratory pressure (PEP) device was added for patients with less severe SIPE.

The aim of this study was to evaluate the feasibility and outcome of prehospital treatment of SIPE with NPPV administered as CPAP by facial mask or by PEP device. Furthermore, we assessed which of the following outcome measures that could reflect successful treatment: peripheral oxygen saturation (Spo_2_), crackles on pulmonary auscultation, findings of pulmonary edema on LUS, and patient-reported respiratory symptoms. In a subgroup with less severe SIPE based on Spo_2_, treatment with CPAP was compared with treatment with a PEP device.

## Study Design and Methods

### Study Design and Population

A prospective observational treatment study was conducted during the open water swimming event Vansbrosimningen in 2017, 2018, and 2019. This yearly event takes place during a 3-day weekend in July in the municipality of Vansbro, Sweden. Approximately 11,000 swimmers complete distances of 1,000 m, 1,500 m, or 3,000 m in cold (16-20 °C) freshwater rivers. The participants represent a broad range of all ages (≥ 10 years), both sexes, competitive swimmers, as well as recreational swimmers. The on-site health care organization includes first aid teams positioned along the riverside and a mobile medical unit (MMU) at the finish area. The MMU consists of a warmed-up military tent and a heated container equipped with four oxygen-driven CPAP stations. Swimmers seeking or being referred to the MMU because of acute onset of cough, dyspnea, or both during or directly after swimming were evaluated for SIPE. Patients arrived within about 10 to 60 min from exiting the water. No treatment was provided before arrival the MMU except for single patients who received oxygen or CPAP during a short ambulance ride to the MMU or used their own asthma medication at the riverside.

All swimmers (≥ 18 years of age) with a diagnosis of SIPE and with indication for acute treatment were eligible for the study. SIPE diagnosis was based on previously published criteria.^2^^,^^19^ LUS findings of pulmonary edema confirmed SIPE diagnosis in 2018 and 2019. In case of missing LUS examination during these years (2018, n = 1; 2019, n = 3) and for all individuals in 2017 (n = 59), clinical diagnostic criteria were used based on Spo_2_ of ≤ 95%, crackles on pulmonary auscultation, or both.^2^^,^^19^ The indication for acute treatment of SIPE with NPPV (CPAP or PEP device) was Spo_2_ remaining at ≤ 95% after initial assessment, persistent respiratory symptoms, or both. Patients who did not receive NPPV treatment on site because of spontaneous recovery during initial assessment, because they recieved β-agonist inhalation only, or because they declined treatment as well as patients who were transferred to hospital because of chest pain were excluded from analysis.

Ethical approval was received from the regional ethical review board in Uppsala, Sweden (Identifiers: 2017/216 and 2017/216/1-2). All individuals gave written informed consent for participation.

### Data Collection and Outcome Measures

**e-Figure 1:**
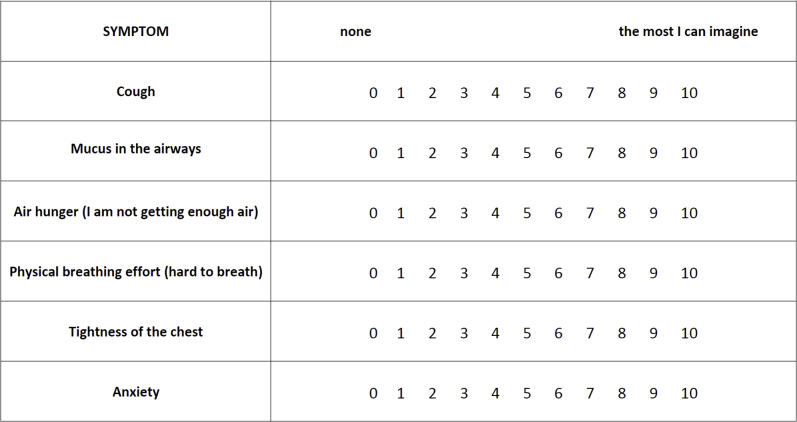


On arrival at the MMU, wetsuits were removed and patients were warmed with blankets. Medical history, clinical parameters, and symptoms were documented. Clinical symptoms were noted by the physician as yes or no for dyspnea, cough, sputum, or hemoptysis. Treatment with CPAP or PEP device was applied as described separately. To evaluate which measures could indicate successful treatment outcome, data for the following four measures were collected: Spo_2_, crackles on pulmonary auscultation, findings of pulmonary edema on LUS, and patient-reported respiratory symptoms. The latter three outcome measures were added over the study period (Table 1). Spo_2_ was measured with a pulse oximeter (Nellcor Oximax N-65; Covidien). Pulmonary auscultation findings were reported by the attending physician as normal breathing sounds, crackles, and other findings. LUS examination was performed by two experienced consultant anesthesiologists as described previously (BK Medical Flex Focus 500 with a curved probe [BK Medical type 8823] of 2-6 MHz; BK Medical AB).^2^^,^^20^ Eight chest regions were scanned for B-lines. For each lung region, a clip of 2 s was recorded, and later reviewed in a masked fashion by the other physician. Bilateral or unilateral presence of two or more positive regions (≥ 3 B-lines) was defined as pulmonary edema.^2^^,^^20^ Six different patient-reported respiratory symptoms were assessed by a numerical rating scale (0-10) (e-Fig 1): two symptoms typically occurring in patients with SIPE (cough and mucus in the airways) as well as four different qualities of dyspnea, modified from the multidimensional dyspnea profile instrument (air hunger, physical breathing effort, tightness of the chest, and anxiety).^21^^,^^22^

**Table 1:** Treatment With CPAP or PEP Device and Outcome Measures Over the 3 Years of the Study

	2017 (n = 37)	2018 (n = 37)	2019 (n = 45)
Treatment properties			
Device (oxygen saturation before treatment)	CPAP (≤ 95%)	CPAP (≤ 95%)	CPAP (≤ 91%) PEP device (≥ 92%)
Treatment cycle: time with device plus rest, min	10 + 10	10 + 10	20 + 10
10-20 min of treatment: No. of treatment cycles	1-2	1-2	1
30-40 min of treatment: No. of treatment cycles	3-4	3-4	2
Outcome measures			
	Spo_2_	Spo_2_	Spo_2_
	. . .	Pulmonary auscultation	Pulmonary auscultation
	. . .	LUS	LUS
	. . .	. . .	Patient-reported respiratory symptoms

LUS = lung ultrasound; PEP = positive expiratory pressure; Spo_2_ = peripheral oxygen saturation.

### Prehospital Treatment With CPAP or PEP Device

**Figure 1:**
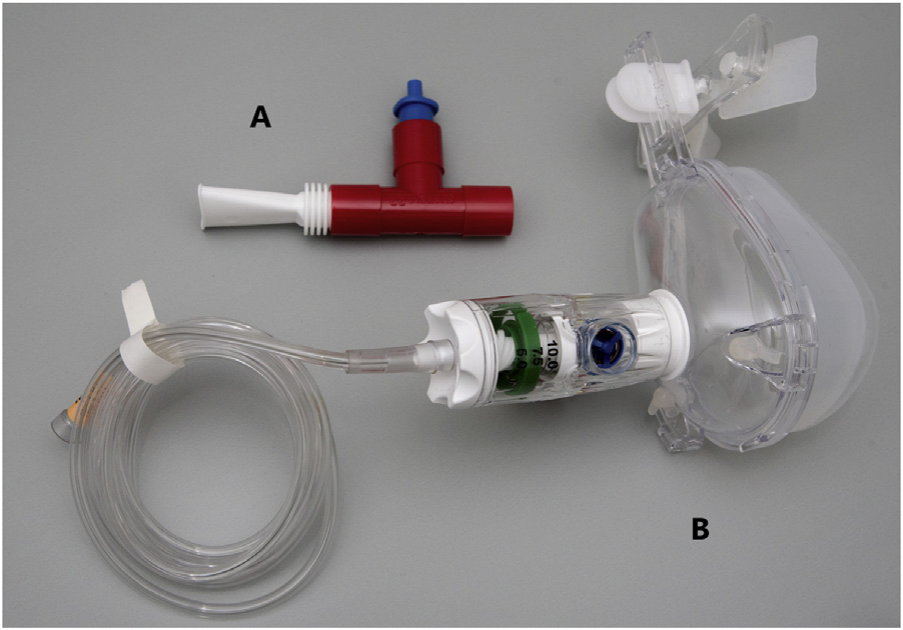
A, B, Photographs showing the devices used for treatment: positive expiratory pressure device (Mini-PEP 3.0 mm; Dolema) (A) and CPAP mask (GO-PAP; Pulmodyne) (B).

Over the years, treatment with CPAP or a PEP device was modified slightly to follow updated guidelines for oxygen treatment and to improve treatment flow on site (Table 1).^23^ Briefly, NPPV was provided by either oxygen-driven CPAP or by a PEP device breathing air (Fig 1). In 2017 and 2018, oxygen-driven CPAP by facial mask was administered to all individuals with indication for NPPV treatment. Here, oxygen flow was set to 10 to 12 L/min to achieve continuous positive pressure of approximately 7 to 8 cm H_2_O, resulting in an inspired oxygen fraction of 40% to 60% (Flow-Safe II; Infiniti Medical). In 2019, patients with saturation of ≤ 91% received oxygen-driven CPAP and patients with saturation of ≥ 92% received treatment with a PEP device. This year, a facial CPAP mask delivering a fixed pressure of 7.5 cm H_2_O by oxygen flow of 10 L/min and stable inspired fraction of oxygen of 30% was used to decrease variation in treatment parameters (GO-PAP; Pulmodyne). The PEP device generated a positive pressure of approximately 7 to 8 cm H_2_O on expiration when patients were instructed to breathe in through the nose and breathe out through the device (Mini-PEP 3.0 mm; Dolema). For all 3 years, adverse events during treatment were noted.

Duration of treatment was set to cycles of 10 min in 2017 and 2018 and was modified to 20 min in 2019. That is, treatment durations of 10 to 20 min corresponded to one or two cycles and treatment of 30 to 40 min corresponded to two to four cycles in the different years (Table 1). Each cycle was followed by a 10-min pause, breathing air, to wash out oxygen before Spo_2_ was measured and further actions were taken. Based on the following clinical evaluation, a new treatment cycle begun or the patient was discharged or transferred to hospital. Discharge criteria consisted of improved Spo_2_, aiming for a stable value of > 95%, together with alleviated subjective respiratory symptoms.^24^ Ambulance transfer to hospital was initiated if discharge criteria were not met after a total treatment duration of 30 to 40 min. A follow-up phone call was made to all patients within 10 days after discharge to register requirement for acute medical care within 24 h after treatment at the MMU.

### Statistical Analysis

Statistical analysis was performed for all individuals treated with CPAP or PEP device and for a subgroup with Spo_2_ of ≥ 92% before treatment. Treatment data were divided based on duration of treatment into three groups: 10 to 20 min, 30 to 40 min, or hospital transfer. An unweighted Kruskal-Wallis test based on pseudoranks was used for comparison of Spo_2_ and respiratory rate in individuals with different treatment duration.^25^ To identify outcome measures that indicated successful treatment on site, those parameters were evaluated separately for individuals discharged from the MMU or transferred to hospital. The Wilcoxon signed-rank test and McNemar test were used for comparison of continuous or nominal data before vs after treatment. The Spearman correlation was used to evaluate the association between Spo_2_ before or after treatment with patient-reported respiratory symptoms. Interrater reliability for LUS was presented by percentage and Cohen’s κ value. Within the subgroup, the Mann-Whitney *U* test and Fisher exact test were used for comparison of continuous or nominal data, respectively. The level of significance of *P* < .05 was adjusted family-wise using the Bonferroni correction for multiple comparisons. For statistical analysis we used IBM SPSS Statistics for Windows version 26.0 (IBM Corp.), the package rankFD version 0.1.0,^26^ and R software version 4.1.2 (R Foundation for Statistical Computing). GraphPad Prism version 8.4.3 software (GraphPad Software) was used for graphic presentation.

## Results

**Figure 2:**
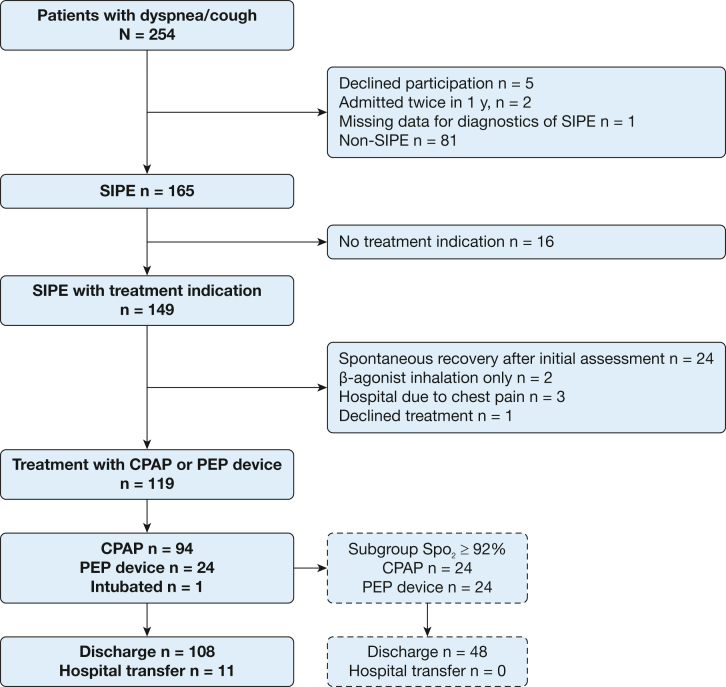
Flow chart showing study progression. PEP = positive expiratory pressure; SIPE = swimming-induced pulmonary edema; Spo_2_ = peripheral oxygen saturation.

Altogether, 32,908 swimmers (≥ 18 years of age) participated in the swimming event from 2017 through 2019.^19^ Of a total of 165 individuals with a diagnosis of SIPE, 119 received treatment with CPAP or a PEP device at the MMU (Fig 2). The mean age of treated swimmers was 48 years, a majority were women, and the median Spo_2_ at admission was 91% (Table 2).

**Table 2:** Background Characteristics and Clinical Findings Before Treatment

Variable	All Individuals (N = 119)	Subgroup With Spo_2_ of ≥ 92%	
		CPAP (n = 24)	PEP Device (n = 24)
Age, y	48 ± 10	46 ± 11	48 ± 10
Sex			
Male	10 (8)	1 (4)	0
Female	109 (92)	23 (96)	24 (100)
Medical history			
Hypertension	18 (15)	6 (25)	1 (4)
Heart disease	5 (4)	0	1 (4)
Asthma	19 (16)	5 (21)	3 (13)
Smoker	1 (1)	0	0
Clinical findings			
Spo_2_, %	91 (88-94)	94 (92-95)	94 (93-95)
Crackles on lung auscultation	111 (93)	24 (100)	21 (87)
Pulmonary edema on LUS^a^	80 (100)^b^	12 (100)	24 (100)
Clinical symptoms			
Dyspnea or cough only	79 (66)	17 (71)	18 (75)
Sputum or hemoptysis	40 (34)	7 (29)	6 (25)
Patient-reported respiratory symptoms, NRS^c^			
Cough	4 (2-7)	—	4 (2-6)
Mucus in the airways	3 (1-7)	—	2 (1-5)
Air hunger	4 (2-7)	—	4 (2-7)
Physical breathing effort	7 (3-8)	—	7 (3-8)
Tightness of the chest	5 (2-7)	—	5 (1-7)
Anxiety	1 (0-4)	—	1 (0-4)

Data are presented as No. (%), mean ± SD, or median (interquartile range). IQR = interquartile range; LUS = lung ultrasound; NRS = numerical rating scale (0-10); PEP = positive expiratory pressure; Spo_2_ = peripheral oxygen saturation; — = no data available.

a Data from 2018 and 2019. All individuals: n = 82; subgroup: CPAP, n = 12; PEP device, n = 24.

b Missing values: n = 2.

c Data from 2019. All individuals: n = 45; subgroup: CPAP, n = 0; PEP device, n = 24.

### Clinical Course of Prehospital Treatment With CPAP or PEP Device

**Figure 3:**
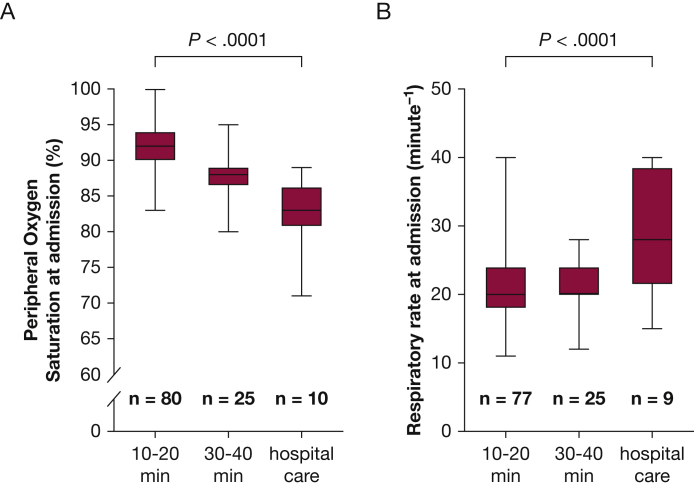
A, B, Box and whisker plots showing treatment duration and corresponding peripheral oxygen saturation (A) and respiratory rate at admission (B) for all individuals. Comparison between groups by the unweighted Kruskal-Wallis test based on pseudoranks (*P* < .0001). Post hoc pairwise comparisons are as follows: *P* < .0001 (10-20 min vs 30-40 min) and *P* = .0005 (30-40 min vs hospital care) (A) and *P* = .833 (10-20 min vs 30-40 min) and *P* = .028 (30-40 min vs hospital care) (B). The level of significance was set to 0.006 after Bonferroni correction for multiple comparisons. Missing values: n = 4 (A) and n = 8 (B).

Of 119 included individuals, 94 were treated with CPAP, 24 were treated with a PEP device, and one required tracheal intubation on site. In total, 108 patients (91%) improved with treatment on site and could be discharged from the MMU. For those, durations of treatment with CPAP or PEP device were 10 min (n = 30), 20 min (n = 52), 30 min (n = 12), and 40 min (n = 13), with missing treatment time for one patient. Transfer to hospital was required for 11 patients (9%), of whom three were treated at the ICU. In addition, one individual discharged from the MMU sought emergency care for respiratory symptoms later the same day. At the ED, this patient showed an Spo_2_ of 100% and normal findings on chest radiography. Longer duration of treatment with CPAP or PEP device was associated with lower Spo_2_ at admission (Fig 3A). Requirement of hospital care was associated with lower Spo_2_ or higher respiratory rate at admission (Fig 3A, 3B), while other clinical findings or background data were similar (data not shown). Three individuals reported adverse events during treatment with CPAP as a feeling of panic (n = 2) and blocked ears (n = 1). No adverse events were reported for treatment with the PEP device. In addition to treatment with CPAP or PEP device, 10 patients (of whom two had previously diagnosed asthma) received inhalation of a β-agonist at the MMU. Furosemide was administered by paramedics to two patients during transfer to hospital. Detailed courses of Spo_2_ during treatment with CPAP or PEP device are presented in e-Table 1.

### Evaluation of Outcome Measures

**Figure 4:**
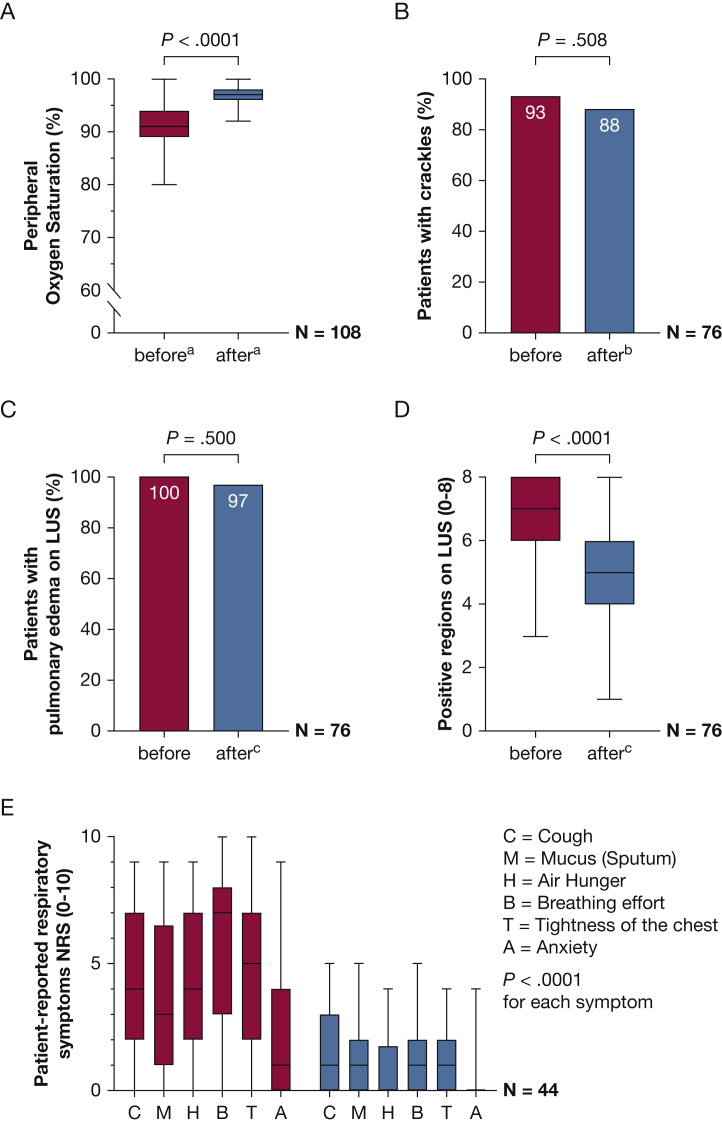
*A-E,* Graphs showing outcome measures before and after treatment with CPAP or PEP device for individuals discharged from the mobile medical unit: peripheral oxygen saturation (percentage) (A), percentage of individuals with crackles on pulmonary auscultation (B), percentage of individuals with pulmonary edema on LUS (C), number of positive regions on LUS (D), and patient-reported respiratory symptoms rated by NRS (0-10) (E). Comparisons of values before vs after treatment using Wilcoxon signed-rank test for continuous data (A, D, E) and McNemar’s test for nominal data (B, C). The level of significance was set to .005 after Bonferroni correction for multiple comparisons. Missing values: n = 2 (A), n = 17 (B), and n = 3 (C). Measures were assessed in different years of the study (Table 1). LUS = lung ultrasound; NRS = numerical rating scale; PEP = positive expiratory pressure.

In patients discharged from the MMU, median Spo_2_ improved from 91% to 95% (*P* < .0001) after the first treatment circle and to 97% after completing treatment with CPAP or PEP device (e-Table 1, Fig 4A). No significant differences were found in the number of patients with crackles on lung auscultation both before (93%) and after (88%) treatment (Fig 4B). Despite a decrease in the absolute numbers of positive regions on LUS after treatment, the remaining number of positive regions on LUS still met the criteria for unilateral or bilateral pulmonary edema in 97% of the individuals (Fig 4C, 4D). For a total of 1,192 regions examined by LUS, the interobserver agreement was 93.8% with a κ value of 0.85 (95% CI, 0.81-0.88). Median numerical rating scale score for each of the six patient-reported respiratory symptoms improved during treatment with CPAP or PEP device (Fig 4E). Of a total of 264 values for patient-reported respiratory symptoms, 246 (93%) improved or were already reported as 0 before treatment. No correlation was found between Spo_2_ and each of the patient-reported respiratory symptoms before, nor after treatment (data not shown). For individuals transferred to hospital, median Spo_2_ increased from 83% to 90% after treatment with CPAP on site, but did not reach stable values of > 95% (missing values, n = 3). Other outcome parameters obtained in 2018 or 2019 were not analyzed for hospitalized patients because of a high proportion of missing values.

### CPAP Compared With PEP Device Subgroup Analysis

**Figure 5:**
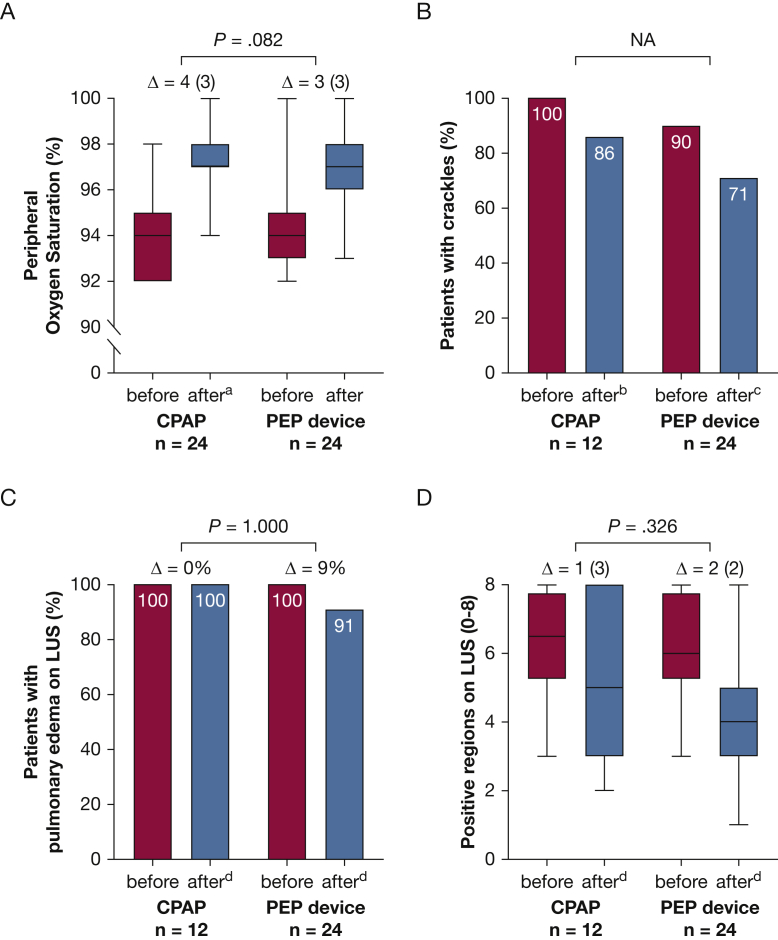
A-D, Graphs showing outcome measures before and after treatment with either CPAP or PEP device in the subgroup with saturation of ≥ 92% before treatment: peripheral oxygen saturation with the difference presented as median (interquartile range [IQR]) (A), percentage of individuals with crackles on pulmonary auscultation (B), percentage of individuals with pulmonary edema on LUS (C), and number of positive regions on LUS with differences presented as median (IQR) (D). The difference before and after treatment was compared between individuals treated with CPAP and PEP device using the Mann-Whitney U test for continuous data (A, D) and Fisher exact test for nominal data (C). No analysis was performed for pulmonary auscultation because of missing values (B). The level of significance was set to .017 after Bonferroni correction for multiple comparisons. Missing values: n = 1 (A), n = 5 (B), n = 3 (C), and n = 1 (D). Δ = median difference between values before and after treatment; LUS = lung ultrasound; NA = not applicable; PEP = positive expiratory pressure.

Patients with Spo_2_ of ≥ 92% before treatment received CPAP in 2017 and 2018 (n = 24) and PEP device treatment in 2019 (n = 24) (Fig 1). Seven patients demonstrated saturation of > 95%, but were treated because of persistent symptoms, three with CPAP and four with PEP device. Treatment duration was 10 to 20 min for all but one individual, who received PEP device treatment for 20 min followed by 20 min of CPAP treatment. All patients with Spo_2_ of ≥ 92% before treatment were discharged from the MMU after treatment. Evaluation of outcome measures showed no difference between treatment with CPAP and PEP device regarding improvement of Spo_2_ and improvement of edema on LUS (Fig 5A, 5C, 5D). Data for pulmonary auscultation were not analyzed because of missing data (Fig 5B). Patient-reported respiratory symptoms were not collected in 2017 and 2018 when CPAP treatment was used.

## Discussion

This study demonstrated that prehospital treatment of SIPE with CPAP or PEP device is feasible on site during a large open-water swimming event. Most patients (91%) could be discharged safely from the on-site MMU after median of 10 to 20 min of treatment with CPAP or PEP device. Moreover, improvement after treatment was reflected by increased Spo_2_ and alleviation of patient-reported respiratory symptoms, whereas LUS findings and lung auscultation remained unchanged. In a subgroup of individuals with Spo_2_ of ≥ 92% at admission, treatment with a PEP device seemed equally efficient to treatment with CPAP.

To the best of our knowledge, this is the first observational study evaluating treatment of SIPE in a large cohort of swimmers. We identified 36 publications regarding treatment of SIPE. These publications were mainly case reports reflecting a general low level of scientific evidence on the subject. Treatment usually consisted of supportive oxygen treatment to reverse acute hypoxia, often combined with diuretics, sometimes also with β_2_-agonist inhalation or NPPV.^5^^,^^12^^,^^13^^,^^27^, ^28^, ^29^, ^30^ In addition, other drugs such as corticosteroids, antibiotics, or nitric oxide were used occasionally.^1^^,^^14^^,^^31^^,^^32^ Rapid initiation of treatment with diuretics requires IV access, which could be technically challenging in hypothermic patients on site. The rationale for β_2_-agonist inhalation can be increased alveolar fluid absorption of pulmonary edema or comorbidity of SIPE and acute asthma.^2^^,^^18^^,^^30^^,^^33^ Simultaneously, reports have been published of patients with SIPE and immersion pulmonary edema with reversible myocardial dysfunction or Takotsubo cardiomyopathy.^12^^,^^34^^,^^35^ In such cases, safety using β_2_-agonist inhalation may be uncertain, and more knowledge about adrenergic activation as a trigger of SIPE is required.^36^ Considering suggested pathophysiologic mechanisms of SIPE, NPPV has the potential to augment the spontaneous resolution of SIPE during warming and rest.^11^ NPPV is thought to accelerate clearance of hydrostatic pulmonary edema by reduction of left ventricular preload and afterload.^37^ Interestingly, only six case reports describing seven patients treated with NPPV for SIPE were identified in addition to our pilot study from 2016.^15^ Six of these patients were treated in hospital, two with CPAP and four with bilevel positive airway pressure.^12^, ^13^, ^14^^,^^16^^,^^17^ Treatment duration with NPPV, reported in three of the hospitalized patients, ranged from 90 min to 6 h.^13^^,^^14^^,^^17^ Only one patient receiving prehospital CPAP treatment, without reported treatment duration, was described by Cochard et al.^9^ In divers with immersion pulmonary edema, a condition similar to SIPE, NPPV has been recommended before pharmacologic treatment.^38^^,^^39^ However, scientific evidence regarding treatment of immersion pulmonary edema in divers is lacking as well.

In our prehospital setting, NPPV for treatment of SIPE administrated as CPAP by facial mask or as a PEP device proved feasible, with short treatment durations (69% of patients were treated for 10-20 min). CPAP was easy to apply with the use of portable oxygen tubes and disposable facial masks. Of note, very few patients experienced adverse events during CPAP treatment. Our data suggested that patients with low peripheral saturation at admission needed longer treatment duration of NPPV and that patients with low Spo_2_ or a high respiratory rate were more likely to require hospital transfer. In contrast, for individuals with less severe SIPE indicated by Spo_2_ of ≥ 92%, treatment with a PEP device seemed to be a suitable alternative. Based on evidence showing drawbacks of hyperoxia, Siemieniuk et al^23^ recommended a target value for Spo_2_ of 90% to 94% for most acute medical conditions. To adjust oxygen administration according to the current guidelines, we introduced PEP device breathing air in 2019.^23^^,^^40^ This first preliminary comparison of PEP device and CPAP indicated that treatment with a PEP device may save resources and may be sufficient in patients with less severe SIPE.

The outcome measures of Spo_2_ and patient-reported respiratory symptoms indicated recovery after treatment with CPAP or PEP device. They separately reflected the objective reversal of hypoxia and the subjective alleviation of dyspnea after treatment. Based on the fact that only one patient discharged from the MMU sought acute medical care within 24 h, these two outcome measures seemed valid to assess readiness for discharge. Of note, patient-reported respiratory symptoms were not available for individuals transferred to hospital. Pulmonary auscultation and LUS examination did not add to the assessment of clinical recovery, but were important for diagnostics of SIPE.^2^ For most patients discharged safely, both pulmonary auscultation and LUS indicated remaining, but clinically insignificant, pulmonary edema. Similarly, LUS has detected subclinical pulmonary edema in asymptomatic triathlon athletes and divers.^41^^,^^42^ Despite findings of pulmonary edema on LUS remaining, we noted a decline in the number of regions with positive findings on LUS after treatment. This partial clearance of edema reflected the gradual recovery also reported in cardiogenic pulmonary congestion.^43^^,^^44^ Altogether, Spo_2_ and patient-reported respiratory symptoms combined could assess treatment indication and readiness for discharge in patients with SIPE.

The main limitation of this prospective observational study is the lack of an untreated control group observed for spontaneous resolution of SIPE to evaluate the efficacy of treatment with CPAP or PEP device. Not enough cases have been described in previous research to present the average course of spontaneous resolution of SIPE within the first hour. Nevertheless, this study was initiated based on our observations of longer treatment times and a higher number of hospital transfers before implementation of NPPV treatment at the MMU. Another limitation to mention is the lack of randomization between treatment with CPAP and PEP device for patients with Spo_2_ of ≥ 92%. Furthermore, LUS was not used to verify SIPE in 2017, and the duration of treatment cycles with NPPV was modified slightly over the study period. All outcome measures were not recorded for each year of the study, and therefore were not available for all included individuals. A large number of pulmonary auscultations (17 of 76) were missing after treatment, most likely because of many patients being treated at the MMU at the same time. Despite these missing data, we decided to include auscultation findings in this descriptive study because dropout analysis could not reveal a systematic loss of data. The patient-reported respiratory symptoms in this study included modified questions based on a previous instrument measuring dyspnea.^21^ These questions have not been validated previously as single items.

In terms of future perspectives, this study provided promising results for the use of CPAP or a PEP device for the treatment of SIPE on site during a large open-water swimming event. Data on treatment course and suitable outcome measures are valuable in designing randomized trials to evaluate the efficacy of treatment with CPAP or PEP device.

## Interpretation

CPAP or PEP device proved feasible and safe for prehospital treatment of SIPE and a vast majority of patients could be discharged from the MMU on site. Spo_2_ and patient-reported respiratory symptoms, but not auscultation of crackles or pulmonary edema on LUS, reflected recovery after treatment. Further randomized controlled trials to confirm the efficacy of CPAP or PEP device for treatment of SIPE are needed.

## Supplementary Data

e-Online Data
