# Supplementary material for: Swimming-Induced Pulmonary Edema: Evaluation of Prehospital Treatment With CPAP or Positive Expiratory Pressure Device
Source: Chest. 2022 Mar 11;162(2):410–20. doi: 10.1016/j.chest.2022.02.054 (PMC9424325; doi:10.1016/j.chest.2022.02.054)
Supplement: e-Online Data [file mmc1.docx]

| **e-Table 1. Peripheral oxygen saturation during treatment with CPAP or PEP-device**. Median saturation and number of individuals within different intervals of oxygen saturation throughout the treatment course: before treatment, after 10-20 minutes treatment and after treatment. Data are presented for all patients (n=119) and for the subgroup with oxygen saturation ≥ 92% before treatment (n=48). | | | | | | | | |  |
| --- | --- | --- | --- | --- | --- | --- | --- | --- | --- |
|  |  | **All individuals** |  |  | **SpO_2_ ≥ 92%** | | | |  |
|  |  | **n=119** |  |  | **CPAP n=24** | | **PEP-device n=24** | |  |
|  | before^a^ | 10-20min treatment^b^ | after^c,e^ |  | before | after^d^ | before | after |  |
| **SpO2 (%); median (IQR)** | 91 (88-94) | 95 (92-97)* | 97 (97-98) |  | 94 (92-95) | 97 (97-98) | 94 (93-95) | 97 (96-98) | |
| **Number of individuals with SpO_2_ (%); n (%)** |  |  |  |  |  |  |  |  |  |
| ≥ 96 | 7 (6) | 36 (45) | 87 (76)^f^ |  | 3 (13) | 20 (87) | 4 (17) | 22 (92) |  |
| 94-95 | 23 (20) | 11 (14) | 17 (15)^f^ |  | 11 (46) | 3 (13) | 12 (50) | 0 |  |
| 92-93 | 18 (16) | 17 (21) | 4 (3) |  | 10 (42) | 0 | 8 (33) | 2 (8) |  |
| 90-91 | 25 (22) | 8 (10) | 3 (3)^g^ |  | 0 | 0 | 0 | 0 |  |
| ≤ 89 | 43 (37) | 8 (10) | 4 (3)^h^ |  | 0 | 0 | 0 | 0 |  |
| missing values: a) n=3; b) n=39 (n=37 in 2017, n=1 2018, n=1 2019); c) n=4; d) n=1 | | | | | | |  |  |  |
| hospital transport: e) n=2 of the individuals with missing values; f) n=1; g) n=3; h) n=4 | | | | | | |  |  |  |
| SpO_2_ = peripheral oxygen saturation , IQR = 25-75 percentile range | | | | |  |  |  |  |  |
| CPAP = continuous positive airway pressure; PEP = positive expiratory pressure  * p<0.001 compared to SpO2 before treatment (Wilcoxon signed-rank test). | | | | | |  |  |  |  |
